# Managing patient deterioration: a protocol for enhancing undergraduate nursing students’ competence through web-based simulation and feedback techniques

**DOI:** 10.1186/1472-6955-11-18

**Published:** 2012-09-28

**Authors:** Simon Cooper, Alison Beauchamp, Fiona Bogossian, Tracey Bucknall, Robyn Cant, Brett DeVries, Ruth Endacott, Helen Forbes, Robyn Hill, Leigh Kinsman, Victoria J Kain, Lisa McKenna, Jo Porter, Nicole Phillips, Susan Young

**Affiliations:** 1 School of Nursing and Midwifery, PO Box 1071, 100 Clyde Rd, Narre Warren, VIC, 3805, Australia; 2 School of Nursing and Midwifery, Monash University, Berwick, VIC, Australia; 3 School of Nursing & Midwifery, The University of Queensland, Salisbury Road, IPSWICH, 4305, Australia; 4 School of Nursing & Midwifery, Deakin University, Burwood Highway, Burwood, 3125, Australia; 5 School of Nursing and Midwifery, Northways Rd, Churchill, VIC, 3842, Australia; 6 School of Nursing and Midwifery, University of Plymouth, Devon, UK; 7 GippsTAFE, Warragul Campus, Warragul, VIC, Australia; 8 School of Rural Health, Monash University, Bendigo, VIC, Australia; 9 School of Nursing and Midwifery, Monash University, Clayton, VIC, Australia; 10 Academic School of Nursing and Midwifery, Northways rd, Churchill, VIC, 3842, Australia

**Keywords:** Education, Nursing, Patient deterioration, Simulation

## Abstract

**Aims:**

To describe a funded proposal for the development of an on-line evidence based educational program for the management of deteriorating patients.

**Background:**

There are international concerns regarding the management of deteriorating patients with issues around the ‘failure to rescue’. The primary response to these issues has been the development of medical emergency teams with little focus on the education of primary first responders.

**Design/Methods:**

A mixed methods triangulated convergent design.

In this four phase proposal we plan to 1. examine nursing student team ability to manage deteriorating patients and based upon these findings 2. develop web based educational material, including interactive scenarios. This educational material will be tested and refined in the third Phase 3, prior to evaluation and dissemination in the final phase.

**Conclusion:**

This project aims to enhance knowledge development for the management of deteriorating patients through rigorous assessment of team performance and to produce a contemporary evidence-based online training program.

## Introduction

In this paper we describe an Australian based funded program of translational research that aims to produce an evidence based education package that will improve nursing student management of deteriorating patients. In Australia the Commonwealth Government has invested in the development of learning and teaching in the higher education sector through the Australian Learning and Teaching Council (ALTC) and more recently the Office for Learning and Teaching (OLT). Established in 2011 the OLT aims to promote excellence in teaching and learning with nationally competitive funding of A$50 million over the next four years [1]. This approach is similar to that in the United Kingdom where the Higher Education Academy was established in 2004 aiming to support research and evaluation in order to enhance students learning experience. These programs aim to improve the quality of teaching and to professionalise the workforce with a strong focus on the transfer of research outcomes into practical and applied educational programs.

In 2011 the MODE-UQGT collaboration received ALTC funding of A$220,000 for three Australian Universities (Monash & Deakin Universities, University of Queensland) and a Further Education partner (GippsTAFE) to produce a sustainable program of education on the management of deteriorating patients. We know that the management of deteriorating patients is poor and when left untreated leads to expensive and often unsuccessful resuscitation procedures [2-5]. In addition, it is acknowledged that non-metropolitan settings have fewer resources for managing deteriorating patients [6,7], communication and referral processes are less well established [4,8], and demands on enrolled and registered nurses have increased. However not enough is known about the educational needs, decision-making strategies and clinical practices of students in order to enhance their future practice. This study aims to address these issues by examining students’ performance in simulated settings, and based on these findings produce on-line evidence based educational materials, assessments, and video recorded interactive scenarios using patient actors. Participants will be able to ‘click on’ actions e.g. taking vital signs and recording electrocardiograms (ECG), whilst records of their performance are retained for feedback.

## Background

Management of deteriorating patients can be influenced by education and past experience [9,10]. In health care contemporary education approaches emphasise the need for active learning [11] and increased use of simulated environments [12-15] to reduce medical errors [14] in settings with high technological, environmental and psychological fidelity [15]. Since 2008 in pilot work with individual nursing students [16,17], midwifery students [18] and Registered nurses working in a hospital setting [19] we have examined patient management in simulated settings using an interventional program known as FIRST^2^ACT (*Feedback Incorporating Review and Simulation Techniques to Act on Clinical Trends*). It was found that participants had good theoretical knowledge but that they often failed to respond appropriately. However, findings also indicated that the educational experience had a significant impact on participants’ reported learning, and significant improvements in patient management skills [20]. A recommendation from these studies was to extend the work to understand how small teams (3 nursing students) respond to clinical emergencies and from these findings produce a web based educational package (FIRST^2^ACT Web). The project will address these objectives in four sequential phases described below. In line with funding criteria the project has received ethical approval and has been independently evaluated by a leading academic experienced in pedagogy of teaching/learning and health care practice. This External Assessor is operating in conjunction with an Internal Assessor with a track record of successful multi-site and multi-national project management. An evaluation plan was developed by the assessors including a risk assessment and staged plan for budget expenditure.

The approach incorporates five components: 1) developing core knowledge; 2) assessment (learning stimulus); 3) simulation; 4) reflective review; and 5) performance feedback [19]. The theoretical underpinning for these stages is the belief that the critical thinking skills necessary for practice are best acquired through experience [21], an approach supported by Experiential Learning Theory [22,23], and the notion of concrete experience and abstract conceptualisation, achieved through activity or reflective observation. In addition, modalities of learning such as visual, visual/verbal, physical (or kinaesthetic), and auditory have been described [24] demanding a variety of teaching approaches to meet students’ learning needs.

Assessment as a stimulus for learning (*for* and *as* learning) contributes to the development of independent learning skills and ongoing professional development [25], encouraging students to “think, decide and act” ( [26] p305). Simulation is defined as “an education technique in which elements of the real world are appropriately integrated to achieve specific goals related to learning or evaluation” ( [27] p75). Clinical simulation, which should always include feedback techniques, can be enacted through mannequins, actors and role play, consolidating theory into practice in a safe environment whilst reducing the pressure on hospital learning environments [28-31]. Simulation-based training increases self-reported knowledge and/or confidence in nursing and medical students [31,32], has benefits over didactic teaching techniques [33], improves outcomes in Objective Structured Clinical Examinations (OSCE) [28], and most importantly positively impacts patient care [34]. Authenticity or fidelity is essential to replicate the clinical environment with ‘true’ representations of patient states.

Reflection and self-assessment are essential for building skills with benefits of video self-review over oral feedback alone, including realistic and explicit perspectives of performance [35]. However, constructive feedback (debriefing) is also essential for the development of knowledge and skills [36] and enables subsequent reflection [37]. These processes of teaching and learning should be linked in a contiguous process to enhance knowledge and improve skill retention.

## Design/Methods

These findings underpin our pilot work and the approach in this project leading to the following research questions.

Research questions:

1. In emergency situations how do undergraduate nursing students’ perform and what are their decision strategies in primary response teams (Phase 1)? Specifically we aim to:

a. Examine participants’ ability to recognise patient deterioration in a simulated environment and establish which clinical cues are most commonly identified and/or missed as signs of deterioration;

b. Identify the relationship between knowledge and skills in the recognition of physiological changes in a simulated environment;

c. Develop an understanding of decision-making processes through student reflection.

2. What impact does the web-based learning program (Phases 2 & 3) (*FIRST*^*2*^*ACT Web*) have on student learning and on their clinical activity (Phase 4)?

These questions will be addressed through four Phases (over two years). The project is designed as an interventional analysis incorporating mixed methods. The mixed methods approach will use a triangulated convergent design [38] aiming to draw together quantitative and qualitative data to inform the development of each phase and the final outcomes of the study.

### Recruitment and ethical approval

Applicable University and Institutional Ethics approval have been obtained for all phases of the study [Lead institution: Monash University Human Research Ethics Committee CF11/3414 -2011001825]. All students were fully informed of the procedures and voluntarily consented to participate in the project.

### Phase 1: understanding team work and decision making(months 1–8)

A mixed methods approach will be used to capture quantitative and qualitative data from three sources:

1. A multiple choice structured knowledge questionnaire (MCQ).

2. Three video recorded team based simulation exercises (OSCEs) to identify performance skills.

3. Video review of the simulation exercises to facilitate reflective review of performance.

Using a triangulated convergent design we will perform separate quantitative and qualitative analysis and then compare the data with matrices of quantitative findings and qualitative themes [39].

### Population

All final-year nursing students studying on a Bachelor of Nursing or combined degree at Monash University, Gippsland Campus (n = 120); Deakin University, Melbourne Burwood Campus (n = 350); and the University of Queensland, Ipswich Campus (n = 100) will be eligible. Total population n = 570. All participants will have completed a standard educational program on emergency care prior to recruitment.

### Sample

A minimum of 100 students will be selected from across the campuses. In our previous studies we have had the following response rates: nursing students (46%), midwifery students (78%), Registered nurses (82%). We therefore anticipate a response rate of greater than 50%.

### Data collection tools

#### Multiple choice questionnaire (MCQ)

Additional file 1 Completed by participants prior to the OSCE and adapted from previously validated instruments [40,41] and verified by a panel of clinical experts. This measure will enable identification of knowledge in relation to skill performance (at OSCEs) and prompts development of learning (i.e. learning through assessment).

#### Simulation exercises (OSCEs)

Additional file 2 All participants will complete three contrasting simulation exercises in their university clinical skill laboratories. Each scenario will be based on common presenting conditions including an acute myocardial infarction, hypovolaemia and chronic obstructive pulmonary disease. Standardised patient actors will be employed to simulate these presenting conditions to enhance the reality of the simulation. These team based scenarios (for groups of three learners, covering 5 groups a day) have been developed from previous work [16-18] and include nominal yes/no ratings of performance that will be rated and verified by two clinicians.

#### Team emergency assessment measure (TEAM)

Additional file 3 In addition to clinical skill OSCE rating, non-technical teamwork skills will be assessed by two clinicians using the valid and reliable teamwork assessment tool TEAM [42]. Using a rating scale of 0–4 this tool includes ratings of leadership, teamwork and task management and a final overall global rating from 1–10. Detailed guidance on how to use TEAM is available from the authors including the training requirements for assessors.

#### Situation awareness global assessment technique (SAGAT)

On the completion of each scenario team leaders will be asked a series of questions to ascertain their understanding of the situation including their awareness, understanding and prediction of future events in relation to each simulation. A rapid questioning technique is required to encourage respondents to give their first ‘gut reaction’ response to each query. Questions were developed by an expert panel using Goal Directed Task Analysis in order to identify core and peripheral situation awareness requirements [43]. Additional file 4 illustrates this process for the acute myocardial infarction scenario. Questions are designed to identify awareness of the patient’s physiological state and to ascertain respondents’ awareness of the wider situation, for example, what the patient is wearing on their wrist and whether suction is available?

#### Video review

Video recordings of participants’ interaction during the simulation exercise will be used as a memory recall service to stimulate the team to provide a audio-recorded reflective account of their decision making, a process known as ‘photo elicitation’ [44,45]. This review will enhance the educative processes and will enable the teaching team to understand decision making processes.

#### Performance feedback and training

Individual constructive feedback from a trained instructor will follow the video review (and the first three scenarios). Written participant feedback/evaluation will be sought after this first phase of training.

### Data analysis

#### Quantitative

Participant demographics, questionnaire, OSCE and TEAM performance will be described with the use of descriptive and inferential statistics. *Qualitative:* Interview and video data will be content analysed to identify common themes [46].

### Phase 2: development of a web-based electronic educational package (months 9–13)

Based on the findings from previous studies and from Phase 1 we will develop an interactive web based educational package which will include a course handbook; assessment tests - Multiple Choice Questionnaires (MCQ) - performed at the start and end of course; scenarios (OSCEs); and feedback techniques. A series of three professionally recorded videoed OSCEs (based on the three Phase 1 scenarios) will be developed depicting professional actors as patients who are deteriorating. Each of the three OSCE videos will run for eight minutes (the approximate response time for a resuscitation team) with patients significantly deteriorating at the midpoint. A range of ‘pop up’ videos will also be recorded to simulate the taking of vital signs, procedural recordings (e.g. 12 lead ECG & intravenous cannulation), and the patients history (spoken by the actor). Using a ‘mouse over’ function participants will be able to click on an action for example, lay the patient flat, give oxygen, or take an ECG. On completion results will be provided with explanations on performance outcomes and, where the pass mark has been reached, a certificate will be provided.

### Phase 3: program implementation (months 14–17)

The final educational package will be made available to applicable nursing lecturers within the MODE-UQGT collaboration. Web-based and hard-copy instructor material will be produced to guide lecturers through the course material. Lecturers un-associated with the project will be recruited to test the transferability of the material. The sample size of 614 students is a whole population sample allowing for an attrition rate of up to 30%. Based on prior data from nursing students who had completed the FIRST^2^ACT multiple choice questionnaire this sample size significantly exceeds the power required to detect a change in knowledge before and after the FIRST^2^ACT *Web* program [n = 33 participants; based upon MCQ Time 1 = 66% v Time 2 = 85% with a power of 80% and a one sided significance level of 0.05.]

The program will be implemented across cohorts of final-year nursing students at Monash University, Gippsland Campus (approximately 120 students); Deakin University, Melbourne Burwood Campus (approximately 350 students); the University of Queensland, Ipswich Campus (approximately 100 students) and one campus of nursing diploma students as GippsTAFE – Morwell (approximately 44 students). [Total students n = 614]. These sites have been selected as they represent students from two levels of training (Bachelor and Diploma) studying in rural (Monash Gippsland, UQ Ipswich) and urban locations (Deakin Burwood). All sites run generic units/modules in acute care – the *FIRST*^*2*^*ACT Web* program will replace specific teaching approaches to patient deterioration without the need to alter unit/module objectives and assessments. In this trial stage of the program assessment outcomes from *FIRST*^*2*^*ACT Web* will not influence final unit/module assessment outcomes.

### Phase 4: evaluation of learning and dissemination strategy (months 17–24)

An evaluation framework will be developed based upon measures of before and after program knowledge (pre-post quasi-experimental) and qualitative evaluations, using Kirkpatrick’s [47] and Clarke’s [48] models for educational evaluation. Both models describe the need to evaluate programs across the spectrum from personal impact on participants to the impact on society, or in this case the clinical impact. In line with these approaches student satisfaction, knowledge, skill gain and workplace (clinical placement) impact will be identified. The following components will be included:

1. At the commencement of the study produce a picture of how the project will work using an ‘intended project logic’ diagram. This exercise will be performed with stakeholders (project team, reference group, lecturers at trial sites, clinical placement co-ordinators and practice educators) in order to focus on the context and objectives of the evaluation e.g. the impact of *FIRST*^*2*^*ACT Web* on learning and teaching. Outcomes from these discussions will guide the evaluation methods which may include the following approaches.

2. Analysis of outcome data including:

a. MCQ scores pre-post course with applicable inferential analysis (e.g. paired t-tests).

b. Paper-based, in-class student evaluations of:

i. Course satisfaction, e.g. relevance, applicability, interest, delivery mode, and descriptive outcomes.

ii. Reflective self reports of specific knowledge levels pre-post course.

c. Ten stakeholder focus groups, based on a stratified sample (seven in Victoria and three in Queensland) to identify perceived impact of the program. Core themes and outcomes will be identified using Miles and Huberman’s stages of data analysis [49].

3. Based on outcomes from the evaluation, update and adapt *FIRST*^*2*^*ACT Web* to meet learning and teaching requirements.

### Dissemination strategy

Outcomes from this project will include a web-based, evidence-based teaching and learning package incorporating innovative simulation techniques. Using a multi-faceted strategy that targets local, national and international interdisciplinary audiences findings and outcomes will be disseminated across clinical and tertiary education sectors and through policy decision makers in patient safety. This will occur through library repositories and peer review publications, conference presentations and workshops, and a web based instructor forum.

## Discussion

This study is part of a national Australian initiative to enhance the quality of learning and teaching in the higher education sector. Guidelines for funding differ somewhat from research funding schemes in that there is a forthright focus on translation of outcomes, dissemination and implementation strategies. This protocol therefore emphasises these aspects through the development of evidence, which informs program development, which in itself is rigorously evaluated and adapted. There are a number of issues associated with a large project of this sort. For example, leadership and management, recruitment strategies and ethical considerations, departmental and university collaboration, technical development and web access issues, and team member succession planning. The project teams early assessment of the risks have raised many of these issues and enabled them to be proactively addressed.

In summary, this project aims to enhance knowledge development through rigorous assessment of team performance and the educative processes of learning through assessment, personal reflective learning, and professional feedback. This will lead to an in-depth understanding of decision making and a contemporary interactive evidence-based online training program. Results and outcomes from the study will be available from 2013.

## Abbreviations

ALTC: Australian learning and teaching council; BP: Blood pressure; CRT: Capillary refill time; ECG: Electrocardiograms; FIRST^2^ACT: Feedback incorporating review and simulation techniques to act on clinical trends; HR: Heart rate; MCQ: Multiple choice questionnaire; MONA: Morphine oxygen, nitrates, asprin; OSCE: Objective structured clinical examinations; OLT: Office for learning and teaching; RR: Respiratory rate; SAGAT: Situation awareness global assessment technique; SpO^2^: Oxygen saturation; TEAM: Team emergency assessment measure.

## Competing interests

The author(s) declare that they have no competing interests.

## Authors’ contributions

SC, FB, TB, RC, FB, BD, RE, HF, HF, LK, LM, JP, NP, SY contributed to the conception and design of this study; SC drafted the manuscript; AB, SC, FB, TB, RC, BD, RE, HF, RH, LK, VK, LM, JP, NP, SY critically reviewed the manuscript. All authors read and approved the final manuscript.

## Pre-publication history

The pre-publication history for this paper can be accessed here:

http://www.biomedcentral.com/1472-6955/11/18/prepub

## Supplementary Material

Additional file 1 **Appendix 1.** Participant demographic form. Click here for file

Additional file 2 **Appendix 2.** Objective Structured Clinical Examinations. Click here for file

Additional file 3 **Appendix 3.** Team Emergency Assessment Measure (TEAM). Click here for file

Additional file 4 **Appendix 4. **Process for development of Situation Awareness (SA) questions. Click here for file
