# Supplementary material for: Managing patient deterioration: a protocol for enhancing undergraduate nursing students’ competence through web-based simulation and feedback techniques
Source: BMC Nurs. 2012 Sep 28;11:18. doi: 10.1186/1472-6955-11-18 (PMC3534359; doi:10.1186/1472-6955-11-18)
Supplement: Additional file 1 — Appendix 1. Participant demographic form. [file 1472-6955-11-18-S1.doc]

Appendix 1

| Participant demographic form | | | | | | |
| --- | --- | --- | --- | --- | --- | --- |
| Your sex? (please tick one) | Female  Male | | | | | |
| Your age? | …………………..Years | | | | | |
| Your course of university of study? (Please tick one) | Bachelor of Nursing  Bachelor of Nursing/Bachelor of Midwifery  Bachelor of Nursing/Bachelor of Public Health and Health Promotion  Bachelor of Nursing/Bachelor of Applied Science (Psychology)  Bachelor of Nursing/Bachelor of Commerce  Diploma of Nursing  Other (please name): ………………………………………………………. | | | | | |
| What year of your course are you currently studying? | Year 1  Year 2  Year 3  Year 4 | In which semester or trimester are you currently  enrolled? (please tick one) | | | Semester 1  Semester 2  Trimester 1  Trimester 2  Trimester 3 | |
| Have you ever worked as an employee in a nursing or healthcare related field (eg., EN, PCA) | No  Yes – If yes, what was your role and how many years did you work in that role? ………………………………………………………………………….  …………………………………………………………………………………….. | | | | | |
| Where have your clinical placements been during your nursing education? (please tick any) | Aged care | |  | General wards | |  |
|  | Community | |  | Mental Health | |  |
|  | Critical / intensive care | |  | Operating Theatre | |  |
|  | Emergency | |  | Rehabilitation | |  |
|  | Other (please specify) | |  | | | |
| Have you ever cared for a patient whose condition suddenly deteriorated such that a medical emergency or Medical Emergency Team (MET) was called? | No  Yes –If yes, what was your role?  Observer  Recorder/scribe  First responder  Calling MET  None  Comments ……………………………………………………………………… | | | | | |
