# Supplementary material for: Managing patient deterioration: a protocol for enhancing undergraduate nursing students’ competence through web-based simulation and feedback techniques
Source: BMC Nurs. 2012 Sep 28;11:18. doi: 10.1186/1472-6955-11-18 (PMC3534359; doi:10.1186/1472-6955-11-18)
Supplement: Additional file 2 — Appendix 2. Objective Structured Clinical Examinations. [file 1472-6955-11-18-S2.doc]

**Appendix 2: Objective Structured Clinical Examinations**

**Scenario 1: Cardiovascular System (Acute Myocardial Infarction)**

*Research Staff:*

- Participants should be asked to arrive dressed as they would for clinical placement. That is, in uniform, hair and jewellery appropriate, note pad, pen, watch, stethoscope, etc..
- Ask participant not to discuss the scenarios with their colleagues until study is complete.
- Ensure demographics form is completed
- Ensure MCQ test is completed
- Ensure microphone/video is correctly placed over the patient actor
- Ask SA questions at the end of the scenario
- Simplify room and monitoring with BP, O^2^ Sats available
- Have an ECG machine available
- Brief ‘newly qualified doctor’ to support appropriately but not to prompt, i.e. they can give drugs and increase infusion rate if requested.
- Run through scenario with participants and ask them to repeat it back.
- Emphasize the need to record observations regularly and verbalise thoughts and actions

**Briefing Notes**

*Nursing student* : You are just starting your shift. There are two other Registered Nurses working on the ward who you can call on for assistance if needed. You also have the support of a junior doctor who will assist and support as required. As your ‘patient’ is an actor you are required to take observations as per normal but results will be revealed by your doctor. The patient is in a quiet side ward.

*The patient:* John is 65 years of age and was admitted a few days ago with cellulitis of his leg for which a course of IV antibiotics has been completed. The IV cannula has been removed and he is due for discharge to home this afternoon. He has just rung the patient call bell. You respond and enter his room. He tells you he has chest pain and points to the centre of his chest. You are the first to respond.

The scenario will be run in ‘real time’. There will therefore be gaps in activity, *(this does not mean you are doing anything wrong).* An observation chart is available for you to document your observation findings. Talk out loud about what you are thinking and doing. You can ask for the patient’s status at any point and you can expose him down to his underwear.

At the end of the simulation you will be stopped and asked about specific aspects of the situation, as you perceive them, at that time. The questions should be answered as rapidly as possible – it is Ok to use your instinct.

**Supporting Doctor - role**

DO NOT PROMPT at any point. Give information as requested after an applicable action, i.e. only indicate the BP or HR after it has been taken. Please rate performance on the following scale during or immediately after each scenario.

Provide the participant with the Inferior acute myocardial infarction ECG after one has been done.

**Patient scenario:**

You are **Mr John Edwards a 65-year-old retired accountant**.

**Moulage** – Cyanosis – i.e. pale/sweaty lips (does the actor have the correct make up?)

*Presenting condition (If asked)*

You were admitted a few days ago for cellulitis of the leg and have been treated successfully with IV antibiotics. About 20 minutes ago you got severe chest pain and you rang the patient buzzer for a nurse.

- **Chest pains and breathlessness**.
- The pain came on gradually and is currently approx **5/10**.
- The onset of **pain was AT REST**. You did not have indigestion.
- The pain was across the front of your chest. It did not radiate anywhere else.
- The pain was accompanied by you feeling generally unwell and breathless. You still feel your breathing is ‘a bit tight’.
- You have had this pain in the past. It **does feel similar to your angina pain.**
- Usually you need to use your GTN approx once every month or so and you have not seen your GP about your angina for the last 8-9 months.

You are anxious and agitated but not aggressive. Your wife is out shopping with her sister and you have been unable to contact them so far.

*Past medical history*

- You are known to have high BP for which you take medication.
- You had a blood test to check your cholesterol last year which was 5.4
- You have had angina for the last three years

*Drug history*

- **Metoprolol** 50mg twice a day (for your BP and angina – you think)
- **Aspirin 100mg** daily
- **Pravastatin 40mg at night (**for your high cholesterol)
- **GTN spray** prn (for your angina when you need it)

*Social history*

- You drink 4 glasses of red wine per day.
- You eat ‘healthily’.
- You smoked 20 cigarettes per day for 25 years but have recently given up
- You have gained about 6kg in weight over the last six months.
- Married to Grace, also retired, with four adult children.
- You don’t exercise specifically but you take your dog for a walk twice a day

*Family history*

- Your father died aged 48 years of a heart attack which is adding to your concerns.

**Decline at 4 minutes (halfway point of scenario)**

- **Rapid increase in chest pains (9/10) and breathlessness (rapid shallow breaths)**
- The pain is **crushing central chest pain** right across the front of your chest. It did not radiate anywhere else.
- You are anxious, agitated and very frightened.

**Participant No = Date=**

| **CVS Chest Pain (Scenario 1)** | | | | | |
| --- | --- | --- | --- | --- | --- |
| **Aprox Time (mins)** | **Observations** | **Action** | **Correct/**  **incorrect** | | ***Points at debrief*** |
| On arrival  1-4 | 5/10  BP 150/95  HR 110 *(if palpated)*  RR 20  CRT – 2 secs  O^2^ Sats 95%  Temp 36.8 | Obtain immediate history  Pain assessment  Record/request obs | Y/N  Y/N  Y/N  Y/N  Y/N  Y/N  Y/N  Y/N | | PQRST (**P**rovoke/Palliation, **Q**uality, **R**adiates, **S**everity, **T**ime) |
|  |  | Investigate current medication usage  Identify other symptoms  Consider non-cardiac causes of chest pain  Aspirin (sub-lingual)  Performed a 12 lead ECG | Y/N  Y/N  Y/N  Y/N  Y/N | | Prescription, over counter, recreational  Dyspnoea, nausea, diaphoresis, neck vein extension  Aortic aneurysm, oesophageal reflux, pneumothorax, musculoskeletal |
|  | | | | | |
| **Patient rapidly deteriorates**  Rapid increase in chest pains (9/10) and breathlessness (rapid shallow breaths) | | | | | |
| 4-7.5 | 9/10  BP 170/95  HR 140  RR 32  CRT – 2 secs  O^2^ Sats 89% *(despite O^2^ if on)* | Pain assessment  Nitrates  Record/request  Obs. | | Y/N  Y/N  Y/N  Y/N  Y/N  Y/N    Y/N | Emphasise systematic ABCs. Time critical |
|  |  | Call for Assistance  Nurses  Doctor  Met call  Position appropriately  Administer 0^2 (non-rebreath)^  Ensure IV cannulation  Blood specimens  Morphine  Assigns tasks to nurses during scenario | | Y/N  Y/N  Y/N  Y/N  Y/N  Y/N  Y/N  Y/N  Y/N | Upright or Semi-recumbent.  **MONA** – **M**orphine, **O**xygen, **N**itrates, **A**sprin  Allocates tasks to nurses, ECG, Vital signs. |
| 7.5 -8 mins? | BP 140/80  HR 120  RR 25  CRT – 2 secs  O2 Sats 93% | **Instructor Note**:  Unless majority of above have been missed indicate these observations and initial stabilisation. | |  | Stabilisation may be temporary |
| End scenario with SA questions | | | | | |

**Scenario 2: Shock - Hypovolaemia**

*Research Staff:*

- Participants should be asked to arrive dressed as they would for clinical placement. That is, in uniform, hair and jewellery appropriate, note pad, pen, watch, stethoscope, etc.
- Ask participant not to discuss the scenarios with their colleagues until study is complete.
- Ensure demographics form is completed
- Ensure MCQ test is completed
- Ensure microphone/video is correctly placed over the patient actor
- Ask SA questions at the end of the scenario
- Simplify room and monitoring with BP, O^2^ Sats available
- Have an ECG machine available
- Brief ‘newly qualified doctor’ to support appropriately but not to prompt, i.e. they can give drugs and increase infusion rate if requested.
- Run through scenario with participants and ask them to repeat it back.
- Emphasize the need to record observations regularly and verbalise thoughts and actions

*Nursing student*: You are just starting your shift. There are two other Registered Nurses working on the ward who you can call on for assistance if needed. You also have the support of a junior doctor who will assist and support as required. As your ‘patient’ is an actor you are required to take observations as per normal but results will be revealed by your doctor. The patient is in a quiet side ward.

A 64 year old otherwise well man is admitted to your ward with abdominal pain. An IV line has been inserted and he has been prescribed 1000mls of IV normal saline over the next 8 hours. No definitive diagnosis has been made but it is not myocardial.

The scenario will be run in ‘real time’. There will therefore be gaps in activity, *(this does not mean you are doing anything wrong).* An observation chart is available for you to document your observation findings. Talk out loud about what you are thinking and doing. You can ask for the patient’s status at any point and you can expose him down to his underwear.

At the end of the simulation you will be stopped and asked about specific aspects of the situation, as you perceive them, at that time. The questions should be answered as rapidly as possible – it is Ok to use your instinct.

**Supporting Doctor - role**

DO NOT PROMPT at any point. Give information as requested after an applicable action, i.e. only indicate the BP or HR after it has been taken. Please rate performance on the following scale during or immediately after each scenario.

**Patient scenario:**

You are **Mr Greg Sephton a 64-year-old retired Engineer**

**Moulage** – Cyanosis – i.e. pale/sweaty, blue lips ears and digits

*Presenting condition (If asked)*

You have had acute abdominal pain in the left lower quadrant since 22.00hrs last night; 6/10 pain score, nil radiation, with rebound tenderness. You have been vomiting and remained nauseated overnight. You presented to ED before being transferred to the surgical unit and have been diagnosed with acute appendicitis waiting for surgery later today. You have an IV cannula insitu with 1000 mL N/saline over 8 hours.

About 20 minutes ago you became sweaty and dizzy, you vomited once then you rang the patient buzzer for a nurse.

- Dizzy light headed, clammy skin and feeling faint.
- The abdominal pain remains at a 5/10

You are anxious and agitated but not aggressive. Your wife has gone down to the cafeteria for a cup of tea and then a walk outside.

*Past medical history*

- Mild Asthma
- Right knee replacement 2000

*Drug history*

- Ventolin prn

*Social history*

- You ride your push bike every weekend with a friend
- Married to Hilda, also retired, with three adult children.

*Family history*

- Your father died aged 48 years of a heart attack

**Decline at 4 minutes (halfway point of scenario)**

- **Rapid increase in abdominal pains (9/10) and breathlessness (rapid shallow breaths) and a rigid abdominal area on palpation.**
- You are anxious, agitated and very frightened.

Participant No = Date=

| **Shock (Scenario 2)** | | | | | | |
| --- | --- | --- | --- | --- | --- | --- |
| **Aprox Time (mins)** | **Observations** | **Action** | **Correct/**  **incorrect** | | | ***Points at debrief*** |
| On arrival  1-4 | BP 95/70  HR 110  RR 19  Temp 37.2  AVPU – Alert  CRT – 2 secs  O2 Sats 95%  5/10 abdo pain | Record / request obs  Obtain immediate history  Pain assessment  Pain relief given  Oxygen  ncrease infusion rate (1L/30min) | Y/N  Y/N  Y/N  Y/N  Y/N  Y/N  Y/N  Y/N  Y/N  Y/N  Y/N  Y/N | | | PQRST pain assessment tool |
| **Patient rapidly deteriorates**  Rapid increase in chest pains (9/10) and breathlessness (rapid shallow breaths) | | | | | | |
| 4-7.5 | BP 75/35  HR 130  RR 25  Temp 37.2  AVPU – Voice  CRT – 5 secs  O2 Sats 89% (despite O2 if on) | Record/ request obs  Call for assistance  Nurses  Doctor  Met Call  Position appropriately  Pain assessment  Request or increase infusion rate (e.g. at least 500mls/5mins)  Administer 0^2 (non-rebreath)^  Ensure IV Cannulation | | Y/N  Y/N  Y/N  Y/N  Y/N  Y/N  Y/N  Y/N  Y/N  Y/N  Y/N  Y/N  Y/N  Y/N  Y/N | Emphasise systematic ABCs. Time critical  Legs elevated | |
| 7.5 -8 mins? | BP 105/75  HR 70  RR 15  Temp 37.2  AVPU - Alert  CRT – 2 secs  O2 Sats 93%  (Despite O2 if on) | **Instructor Note**:  Unless majority of above have been missed indicate these observations and initial stabilisation. | | Y/N  Y/N  Y/N  Y/N  Y/N  Y/N  Y/N |  | |
| End scenario with SA questions | | | | | | |

**Scenario 3: Respiratory system (Chronic Obstructive Pulmonary Disease)**

*Research Staff:*

- Participants should be asked to arrive dressed as they would for clinical placement. That is, in uniform, hair and jewellery appropriate, note pad, pen, watch, stethoscope, etc.
- Ask participant not to discuss the scenarios with their colleagues until study is complete.
- Ensure demographics form is completed
- Ensure MCQ test is completed
- Ensure microphone/video is correctly placed over the patient actor
- Ask SA questions at the end of the scenario
- Simplify room and monitoring with BP, O^2^ Sats available
- Have an ECG machine available
- Brief ‘newly qualified doctor’ to support appropriately but not to prompt, i.e. they can give drugs and increase infusion rate if requested.
- Run through scenario with participants and ask them to repeat it back.
- Emphasize the need to record observations regularly and verbalise thoughts and actions

*Nursing student*: You are just starting your shift. There are two other Registered Nurses working on the ward who you can call on for assistance if needed. You also have the support of a junior doctor who will assist and support as required. As your ‘patient’ is an actor you are required to take observations as per normal but results will be revealed by your doctor. The patient is in a quiet side ward.

*The patient:* Brian is a 65 year old who has just arrived on the ward for a TURP later this afternoon. He has rung his buzzer complaining of shortness of breath, you are the first nurse to attend.

The scenario will be run in ‘real time’. There will therefore be gaps in activity, *(this does not mean you are doing anything wrong).* An observation chart is available for you to document your observation findings. Talk out loud about what you are thinking and doing. You can ask for the patient’s status at any point and you can expose him down to his underwear.

At the end of the simulation you will be stopped and asked about specific aspects of the situation, as you perceive them, at that time. The questions should be answered as rapidly as possible – it is Ok to use your instinct.

**Supporting Doctor - role**

DO NOT PROMPT at any point. Give information as requested after an applicable action, i.e. only indicate the BP or HR after it has been taken. Please rate performance on the following scale during or immediately after each scenario.

**Patient scenario**

You are Mr Brian Reed, 65 years of age, employed as a storeman at a local Bunnings store. You are married with two children; 20 and 22 years). You have a fear of doctors and hospitals and rarely seek treatment.

You are 175 cm tall and a little overweight

**Presenting complaint (if asked)**

You have been having episodes of frequent urination and burning. You have an enlarged prostate and your GP has referred you for a TURP. In the last day or so you have developed a productive cough and have been breathless at rest. (*During the scenario short of breath, cough, wheeze, fast breathing, use accessory muscles – lift shoulders up and down)*

**History of presenting complaint**

- Short of breath for many years but worsening over the last few days
- You have had a cough since a recent cold that will not go away.

If asked:-

- The cough produces thick sticky yellow phlegm and can be described as harsh and chesty.
- Your mum said you were always a “wheezy, snotty child”
- When breathless you feel you can’t fill up your lungs with air
- In the past, the symptoms have subsided within 2-3 minutes if you stop what you are doing
- You know its not your heart but you are frightened it may be lung cancer because of your smoking
- **You have not coughed up any blood ***
- **You have no chest pain, no leg pain and no periods of immobility. You have not travelled recently***
- **Your legs have not been swollen***
- **You voice has not been hoarse***

** Key negative features*

**Past medical history**

- Tonsils and adenoids removed as a child
- **If asked**
  - **you do not suffer with hay fever**
  - **You have never had eczema**

**Drug history**

- No current prescribed medication
- If asked –
  - your wife insists that you take a daily multivitamin
  - you have no known drug allergies
  - you get an itchy rash if you sit on newly mown grass

**Social history**

- You started smoking 50 years ago (now 20 a day)
- You drink 5 cans of beer most weekends
- You are a little overweight and eat a lot of take-away meals.
- Married to Lyn with two children. Your family are fit and well
- You don’t take regular exercise but feel your job keeps you fit.
- You have not been exposed to occupational pollutants at work

**Family history**

- Both your parents are alive and well but your father has high blood pressure
- You have one younger sister who is asthmatic and has eczema

**Moulage** – cyanosis – blue lips

**At 4 minutes - patient rapidly deteriorates: *short of breath, cough, wheeze, fast breathing, use accessory muscles – lift shoulders up and down***

Participant No = Date=

| **Respiratory (Scenario 3)** | | | | |
| --- | --- | --- | --- | --- |
| **Aprox Time (mins)** | **Observations** | **Action** | **Correct/**  **incorrect** | ***Points at debrief*** |
| On arrival  1-4 | BP 135/95  HR 100  RR 28  CRT – 2 secs  O^2^ Sats 90%  Temp 38.8 | Obtain immediate history  Record/request obs | Y/N  Y/N  Y/N  Y/N  Y/N  Y/N  Y/N | Discuss ‘Blue Bloater’  (Chronic bronchitis)  Dyspnoea, cyanosis, cough, wheeze |
|  | Bilateral basal  Wheeze and course crackles | Investigate current medication usage  Identify symptoms/negative features  Auscultate  Chest  Administer Oxygen | Y/N  Y/N  Y/N  Y/N | Prescription, over counter, recreational  Heart failure, blood expectoration, leg pain/oedema, travel.  Discuss O_2_ levels – objective 90%sats etc |
|  | | | | |
| **Patient rapidly deteriorates** *short of breath, cough, wheeze, fast breathing, use accessory muscles – lift shoulders up and down* | | | | |
| 4-7.5 | BP 170/110  HR 150  RR 35 +accessory  ++ wheeze  CRT – 2 secs  O^2^ Sats 82% *(despite O^2^ if on)* | Record/request  Obs. | Y/N  Y/N  Y/N  Y/N    Y/N | Emphasise systematic ABCs. Time critical  Aim for O2 sats of 90% |
|  |  | Call for assistance  Nurses  Doctor  Met Call  Position appropriately  Request IV cannulation  Request nebuliser  (beta2-agonist, anticholinergics  Consider anti-biotics  Consider non-invasive vent. | Y/N  Y/N  Y/N  Y/N  Y/N  Y/N  Y/N  Y/N | *Orthopnoeic position* |
| 7.5-8 mins? | BP 140/80  HR 145  RR 32  CRT – 2 secs  O2 Sats 89% | **Instructor Note**:  Unless majority of above have been missed indicate these observations and initial stabilisation. |  | Stabilisation may be temporary |
| End scenario with SA questions | | | | |
