# Supplementary material for: Managing patient deterioration: a protocol for enhancing undergraduate nursing students’ competence through web-based simulation and feedback techniques
Source: BMC Nurs. 2012 Sep 28;11:18. doi: 10.1186/1472-6955-11-18 (PMC3534359; doi:10.1186/1472-6955-11-18)
Supplement: Additional file 3 — Appendix 3. Team Emergency Assessment Measure (TEAM). [file 1472-6955-11-18-S3.doc]

**Appendix 3: Team Emergency Assessment Measure (TEAM)
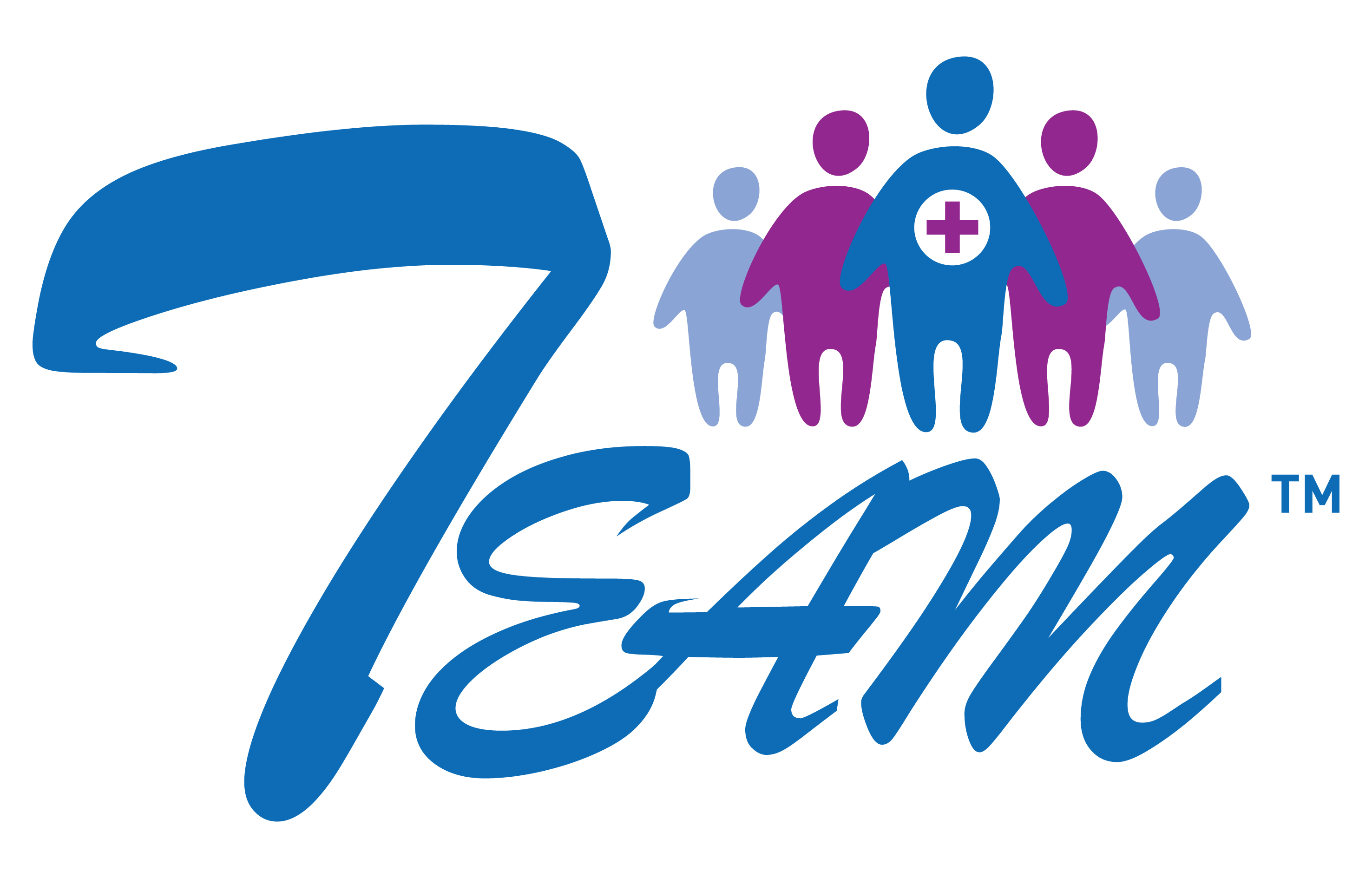
**

| **Introduction** |
| --- |

This non- technical skills questionnaire has been designed as an observational rating score for valid, reliable and feasible ratings of emergency medical teams (e.g. resuscitation and trauma teams). The questionnaire should be completed by expert clinicians to enable accurate performance rating and feedback of leadership, team work, situation awareness and task management. Rating prompts are included where applicable. The following scale should be used for each rating:

| Never/Hardly ever | seldom | About as often as not | Often | Always/Nearly always |
| --- | --- | --- | --- | --- |
| 0 | 1 | 2 | 3 | 4 |

| **Team Identification** |
| --- |

Date: Time: Place: Team Leader: Team:

| **Leadership: it is assumed that the leader is either designated, has emerged or 0 1 2 3 4**  **is the most senior - if no leader emerges allocate a ‘0’ to question 1 and 2.** |
| --- |
| **1.The team leader let the team know what was expected of them through**  **direction and command** |
| **2. The team leader maintained a global perspective**  *Prompts: Monitoring clinical procedures and the environment? Remaining ‘hands off’*  *as applicable? Appropriate delegation.* |
| **Team Work: ratings should include the team as a whole i.e. the leader and the team 0 1 2 3 4**  **as a collective (to a greater or lesser extent).** |
| **3. The team communicated effectively**  *Prompts: Verbal, non-verbal and written forms of communication?* |
| **4. The team worked together to complete the tasks in a timely manner** |
| **5. The team acted with composure and control**  *Prompts: Applicable emotions? Conflict management issues?* |
| **6. The team morale was positive**  *Prompts: Appropriate support, confidence, spirit, optimism, determination?* |
| **7. The team adapted to changing situations**  *Prompts: Adaptation within the roles of their profession?*  *Situation changes: Patient deterioration? Team changes?* |
| **8. The team monitored and reassessed the situation** |
| **9. The team anticipated potential actions**  *Prompts: Preparation of defibrillator, drugs, airway equipment?* |
| **Task Management: 0 1 2 3 4** |
| **10. The team prioritised tasks** |
| **11.The team followed approved standards and guidelines**  *Prompt: Some deviation may be appropriate?* |
| **Overall: 1 2 3 4 5 6 7 8 9 10** |
| **12. On a scale of 1-10 give your global rating of the team’s**  **non-technical performance** |

**Comments:________________________________________________________________________**

**________________________________________________________________________________**
