# Supplementary material for: Managing patient deterioration: a protocol for enhancing undergraduate nursing students’ competence through web-based simulation and feedback techniques
Source: BMC Nurs. 2012 Sep 28;11:18. doi: 10.1186/1472-6955-11-18 (PMC3534359; doi:10.1186/1472-6955-11-18)
Supplement: Additional file 4 — Appendix 4. Process for development of Situation Awareness (SA) questions. [file 1472-6955-11-18-S4.doc]

**Appendix 4: Process for development of Situation Awareness (SA) questions**

**Goal Task Analysis (Acute Myocardial Infarction)**

**Key Goal**

Resuscitation

**Sub Goal**

Primary Stabilisation/Resuscitation (first 8 minutes)

**SAGAT Queries**

**Physiological Perception**

What is the BP at the moment?

What is the HR at the moment?

What is the RR at the moment?

**Global Situation Perception**

Is suction available?

What’s on the patient’s wrist?

What was on the wall near the patient?

**Comprehension**

Is the patient adequately oxygenated?

What is wrong with this patient?

**Projection**

If condition does not improve, what will happen to the HR?

If condition does not improve, what will happen to the BP?

What investigations may be required?

What medications may be required?

**Key Decisions**

What is the patients’ status (observations)?

Is assistance required?

What is the differential diagnosis?

What equipment is required?

What responses are required to the observations?

How should the patient be stabilised?

**SA Requirements**

Visual assessment e.g. Respiratory Rate (RR) and global overview?

Physiological monitoring e.g. Blood Pressure (BP), Heart Rate (HR), Temperature, Capillary Refill Time (CRT)?

Awareness of the need for assistance?

Observation/indicators of pain?

Awareness of heart rhythm?

Awareness of equipment requirements?

Awareness of applicable actions (e.g. analgesia)?

Awareness of requirements for patient stabilisation e.g. Morphine, Oxygen, Nitrates Asprin (MONA)?

**Situation Awareness (questions, answers and responses)**

**Cardiac Scenario (Acute Myocardial Infarction)**

| **Question** | **Answer** | **Right** | **Wrong** |
| --- | --- | --- | --- |
| What medications may be required? | 2 of- Morphine, Nitrates, Asprin |  |  |
| What is the HR at the moment? | 140 or 120 |  |  |
| Is the patient adequately oxygenated (O^2^ saturation)? | NO - 89% - 93% |  |  |
| What is on the patient’s wrist? | A friendship band |  |  |
| What investigations may be required? | 2 of -12 lead ECG, Bloods (cardiac enzymes), Chest XRay |  |  |
| What was on the wall near the patient? | Childs drawing |  |  |
| If condition does not improve, what will happen to the HR initially? | Increase |  |  |
| What is wrong with the patient | Acute Myocardial Infarction |  |  |
| What is the BP at the moment? | 170/95  Or 140/80 |  |  |
| What is the respiratory rate at the moment? | 32 or 25 |  |  |
| Is suction available? | Yes |  |  |
| If condition does not improve, what will happen to the BP initially? | Increase then decrease |  |  |
